# Renal toxicity of targeted therapies for renal cell carcinoma in patients with normal and impaired kidney function

**DOI:** 10.1007/s00280-021-04260-y

**Published:** 2021-03-25

**Authors:** Łukasz Mielczarek, Anna Brodziak, Paweł Sobczuk, Maciej Kawecki, Agnieszka Cudnoch-Jędrzejewska, Anna M. Czarnecka

**Affiliations:** 1 Department of Experimental and Clinical Physiology, Laboratory of Centre for Preclinical Research, Medical University of Warsaw, Warsaw, Poland; 2 Department of Oncology and Radiotherapy, Maria Sklodowska-Curie National Research Institute of Oncology, Warsaw, Poland; 3 Department of Soft Tissue/Bone Sarcoma and Melanoma, Maria Sklodowska-Curie National Research Institute of Oncology, Warsaw, Poland; 4 Department of Experimental Pharmacology, Mossakowski Medical Research Centre Polish Academy of Sciences, Warsaw, Poland

**Keywords:** Renal cell cancer, TKI, MTORi, Immune checkpoint inhibitors, Renal insufficiency, Nephrotoxicity

## Abstract

The introduction of novel targeted therapies during the last 2 decades has led to a significant improvement in patients' clinical outcomes with renal cell carcinoma. However, this improvement came at the price of a whole new spectrum of adverse events, including renal toxicity. Systemic treatment of patients with kidney neoplasms who often present with impairment of kidney function, even prior to treatment, poses an increasing diagnostic and therapeutic challenge for clinicians. Common lifestyle-related comorbidities, i.e., hypertension and diabetes, may contribute to further impairment of kidney function. The lack of official guidelines and the exclusion of patients with reduced kidney function from the clinical trials of recently approved drugs complicate the issue even further. Early detection and correct management of renal toxic effects are crucial to preserve kidney function and ensure the optimal administration of life-prolonging therapies. This review presents detailed information on the renal toxicities of three groups of drugs commonly used in renal cell carcinoma treatment: tyrosine kinase inhibitors, mammalian target of rapamycin inhibitors, and immune checkpoint inhibitors. We outline the incidence and underlying mechanisms of renal adverse effects with a focus on patients on renal replacement therapy, as well as present suggestions for their management.

## Introduction

Renal cell carcinoma (RCC) is the 6th most common cancer type in men and the tenth most common in women, accounting for 5% and 3% of all cancer diagnoses, respectively. It remains one of the most lethal urological malignancies [1]. With growing incidence rates, mostly in high-income countries, RCC arises as an important diagnostic and therapeutic challenge. Common lifestyle-related diseases, i.e., hypertension and diabetes, are independent risk factors of RCC development, and, therefore, it is not surprising that hypertensive nephrosclerosis and diabetic nephropathy are common in RCC patients [2–4]. A history of kidney disease is an independent risk factor of RCC (HR 2.58, 95% CI 1.21–5.50) [2]. Approximately 26% of RCC patients have chronic kidney disease (CKD) even before RCC treatment [5]. Patients with end-stage renal disease (ESRD) requiring long-term dialysis or after renal transplantation are at a higher risk of developing RCC—0.3% and 0.7%, respectively [6, 7], compared to approximately 0.005% in the general population [8]. However, despite RCC's higher incidence in ESRD patients, tumors in this population exhibit favorable clinical and pathological features [9, 10]. RCCs in native kidneys of ESRD patients are usually asymptomatic, small, low stage, low grade, and non-metastatic at diagnosis [9]. These differences may be attributed to increased surveillance and the distinct pathophysiology of these tumors, including unique histopathological subtypes such as acquired cystic disease-associated RCC and clear cell papillary RCC [11].

Despite improvements in renal tumors surgery, even patients without previously diagnosed CKD may develop renal insufficiency after RCC resection. New-onset CKD secondary to surgery develops more often in patients undergoing radical nephrectomy than partial nephrectomy, with various rates reported by studies (55.7–70% vs. 6.2–17.4%, respectively) [5, 12]. RCC's surgical treatment may also result in acute kidney injury (AKI) in up to 5% of cases [13]. Patients presenting with sporadic bilateral and/or multifocal renal tumors represent a unique population, accounting for up to 5% of all RCC patients [14]. Despite the introduction of nephron-sparing surgery, a substantial group of patients in this population needs to be treated with bilateral nephrectomy and subsequently develops renal insufficiency that must be treated with dialyzes. Surgical resection is a curative procedure in most localized RCC cases, but 20–40% of the patients will ultimately develop distant metastases and require systemic treatment in the first 5 years after primary tumor surgery [15]. The last few decades brought tremendous changes to the therapeutic landscape of metastatic RCC, with the development of numerous tyrosine kinase inhibitors (TKIs), mammalian target of rapamycin inhibitors (mTORis), and immune checkpoint inhibitors (ICPIs). This progress has significantly improved RCC patients' clinical outcomes, at the price of a whole new spectrum of adverse events (AEs), including renal toxicities. Because of the many links between cancer, kidneys, and drug metabolism, nephrologists should become aware of new anticancer drugs' potential nephrotoxicity and be actively involved in certain aspects of cancer care. Emergence of a new subspecialty, onconephrology, underlines the need for a close collaboration between oncologists and nephrologists [16]. In this paper, we comprehensively review renal toxicities associated with the aforementioned classes of drugs used in the treatment of RCC from an epidemiological, pathophysiological, and clinical point of view. Additionally, we summarize recommendations and underline differences in systemic treatment between patients with CKD and ESRD undergoing hemodialysis.

## Renal toxicities of tyrosine kinase inhibitors

### Incidence

TKIs are one of the most commonly used groups of drugs in RCC treatment and include sunitinib, pazopanib, sorafenib, axitinib, and cabozantinib. All abovementioned drugs are multitargeted TKIs, which means that they interfere with the activity of more than one family of receptor tyrosine kinases, including vascular endothelial growth factor receptor (VEGFR), platelet-derived growth factor receptor (PDGFR), fibroblast growth factor receptor (FGFR), and epidermal growth factor receptor (EGFR), and all share a similar structure. Additionally, sorafenib is a potent inhibitor of serine/threonine-protein RAF kinases. Their shared mechanism of action contributes to their similar side effects. The most common AEs involve gastrointestinal, skin, bone marrow, and cardiovascular system toxicities. Renal toxicities are also frequently reported; however, data regarding their exact rate are inconsistent (Table 1).

**Table 1:** Renal toxicities in major TKI trials

Trial	Drug	Number of patients	Renal related toxicities			
			Type	Overall, any grade (%)	Grades 3–4/SAE (%)	Refs.
Renal EFFECT Phase II, 1st line NCT00267748	Sunitinib	147	Creatinine increase	69	2	[104]
Phase II, 2nd line NCT00077974	Sunitinib	106	Creatinine increase^a^	8.49	ND	[105]
			Acute renal failure^a^	2.83	2.83	
Phase III, 1st line Registration trial NCT00083889	Sunitinib	375	Creatinine increase^a^	66	ND	[17, 18]
			Renal failure^a^	1.07	1.07	
			Acute renal failure^a^	1.07	1.07	
			Hematuria^a^	1.33	1.33	
			Nephrotic syndrome^a^	0.27	0.27	
COMPARZ (pazopanib vs sunitinib) Phase III, 1st line NCT00720941	Sunitinib	553	Creatinine increase	46	2	[24]
			Proteinuria^a^	14	0.18	
			Acute renal failure^a^	1.62	1.64	
			Renal failure^a^	0.73	0.73	
			Hematuria^a^	0.18	0.18	
RECORD-3 trial Phase III (sunitinib vs everolimus 1st or 2nd line) NCT00903175	Sunitinib 1L, everolimus 2L/everolimus 1L, sunitinib 2L	231/238	Creatinine increase	11/13	2/3	[106]
Phase II treatment-naive or post-cytokine NCT00244764	Pazopanib	225	Creatinine increase	32	< 1	[107]
Phase II, post-cytokine NCT00731211	Pazopanib	55	Proteinuria	44	13	[108]
			Renal failure	4	4	
Phase III, treatment-naive or post-cytokine NCT00334282	Pazopanib	290	Proteinuria	10	2	[109, 110]
			Hematuria^a^	0.24	0.34	
			Acute kidney injury^a^	0	0	
COMPARZ (pazopanib vs sunitinib) Phase III, 1st line NCT00720941	Pazopanib	554	Creatinine increase	32	1	[24]
			Proteinuria^a^	18	< 1	
			Acute renal failure^a^	< 1	< 1	
			Hematuria^a^	0.36	0.36	
			Renal failure^a^	< 1	< 1	
TARGET Phase III, 2nd line NCT00073307	Sorafenib	451	Renal failure^a^	1.77	1.77	[111, 112]
INTORSECT Phase III, post-cytokine NCT00474786	Sorafenib	252	Creatinine increase^a^	12.85	ND	[113]
			Renal failure	1.61	1.61	
			Acute renal failure^a^	0.4	0.4	
Phase II, post-cytokine NCT00076011	Axitinib	52	Hematuria^a^	9.62	1.92	[114]
			Acute renal failure^a^	1.92	1.92	
Phase II, post-cytokine NCT00569946	Axitinib	64	Proteinuria	63	9	[115]
			Hematuria^a^	1.56	1.56	
AXIS Phase III, 2nd line NCT00678392	Axitinib	361	Creatinine increase	55	0	[23, 116]
			Proteinuria	13	3	
Phase III, 1st or 2nd line NCT00920816	Axitinib	192 (135 Asian pts)	Creatinine increase (data only for Asian pts)	39.2	0.8	[117–119]
			Proteinuria (data only for Asian pts)	20.7	5.2	
CABOSUN Phase II, 1st line NCT01835158	Cabozantinib	79	Creatinine increase	25	3	[120–122]
			Hematuria^a^	2.56	2.56	
			Proteinuria^a^	7.69	1.28	
METEOR Phase III, post-cytokine NCT01865747	Cabozantinib	330	Proteinuria	12	2	[123, 124]
			Creatinine increase	5	< 1	

*ND* no data, *SAE* serious adverse events

^a^Data from clinicaltrials.gov registry

Most TKI-related renal AEs include mild renal injury, defined as a clinically insignificant serum creatinine elevation that develops in up to 70% of patients receiving sunitinib [17–20]. Clinically important renal insufficiency may develop in 7.7% to 33% of patients treated with sunitinib [21, 22]. In the AXIS trial, deterioration of kidney function (defined as creatinine clearance elevation) was observed in 41% of patients treated with axitinib and in 55% of patients treated with sorafenib during the first-line treatment; however, a clinically significant increase of creatinine clearance (grades 3 and 4 according to CTCAE version 3.0) was extremely rare in case of sorafenib (< 1%) and absent in patients receiving axitinib [23]. Pazopanib treatment is associated with a similar prevalence of kidney function deterioration, as observed in the COMPARTZ trial, where increased creatinine levels were present in 32% of patients [24]. Nevertheless, clinically significant kidney function impairment was extremely rare, with < 1% of acute kidney failure cases in this trial. In cabozantinib trials, i.e., CABOSUN and METEOR, 5% and 25% of patients demonstrated elevated creatinine levels, respectively (Table 1). In a retrospective analysis of Japanese patients treated with sunitinib, sorafenib, or axitinib in first, second, or third line, deterioration of kidney function was documented, however, with no significant differences between creatinine clearance at baseline and that at the end of therapy between patients receiving different lines of treatment [25].

Another side effect related to renal function, proteinuria, may develop in up to 63% of patients undergoing anti-VEGF therapy, depending on the type of drug (Table 1). According to a recent meta-analysis of newly approved VEGFR-TKIs (regorafenib, vandetanib, cabozantinib, lenvatinib, axitinib), the risk of developing high-grade proteinuria event was significant for patients with RCC [26]. In the course of nephrotic syndrome, heavy proteinuria may be seen in up to 6.5% of patients treated with anti-VEGF therapies [27].

### Mechanisms of nephrotoxicity

Renal damage induced by TKIs can present with hypertension and proteinuria. In patients receiving anti-VEGF therapy, thrombotic microangiopathy (TMA) is the most common histopathological finding [28]. Cases of mesangioproliferative glomerulonephritis, cryoglobulinemic glomerulonephritis, extracapillary proliferative glomerulonephritis [29], and immune complex-mediated focal glomerulonephritis [30] have also been reported. Patients receiving TKI’s may also experience nausea, vomiting, and diarrhea, which can cause dehydration and, in serious cases, contribute to prerenal kidney failure [31]. The mechanisms of renal toxicity on the molecular level are complex. TKIs activate the endothelin-1 system and modulate the renin–angiotensin system, resulting in hypertension and microvascular dysfunction [32].

Moreover, podocytes—cells forming the filtration barrier in renal glomeruli—are involved in proteinuria's pathophysiology. Podocytes abundantly express VEGF and its receptors, maintaining these cells' physiological function and glomerular filtration membrane integrity. TKIs, by interfering with VEGF receptors' activity on the podocytes, impair the glomerular filtration barrier and, consequently, induce proteinuria and reduced glomerular filtration rate [33]. Some studies suggest that the TKI-mediated podocyte injury might be facilitated by tyrosine phosphorylation of nephrin, a protein expressed in podocytes critical for maintaining the integrity of the filtration barrier [34]. The loss of normal podocyte fenestration results in microvascular injury, capillary thrombosis, and renal glomeruli sclerotization, which may cause TMA. Affected patients develop proteinuria, hypertension, anemia, thrombocytopenia, and eventually renal failure. Proteinuria as a side effect of TKIs is not only caused by increased glomerular permeability. Still, it is also secondary to increased intraglomerular pressure that is an effect of arterial hypertension, another TKI side effect. Even though hypertension and proteinuria often occur simultaneously, it is not clear whether both of these side effects occur independently as an effect of VEGF blockage, or one of them is secondary to the other. Moreover, TKIs may induce acute renal failure through toxic injury of renal tubules or tumor lysis syndrome [33, 34].

### Management

TKI-induced hypertension has been well documented in several studies as a predictor of favorable prognosis in metastatic RCC (mRCC) patients [20, 35]. However, data regarding TKI-induced renal function impairment as a prognostic factor for RCC patients are limited. In a retrospective analysis of Korean patients receiving sunitinib, the incidence of renal AEs, namely proteinuria, was associated with longer progression-free survival (PFS) [21]. A pooled secondary analysis of patients with mRCC treated with pazopanib and sunitinib in phase III randomized clinical trials showed that proteinuria, particularly grades 3/4, was associated with improved overall survival (OS) (HR 0.53, 95% CI 0.30–0.92) [36]. In a previous analysis, the development of new-onset hypercreatinemia during TKI treatment was related to a more favorable prognosis in OS and PFS [20]. These results suggest that the presence of renal AEs during TKI treatment should not necessitate treatment discontinuation, especially when the side effects are moderate. There are no evidence-based recommendations on the treatment of TKI-associated renal AEs. Most patients with proteinuria are treated with renin–angiotensin system blockers (angiotensin-converting enzyme inhibitors or angiotensin II receptor blockers); however, no interventional studies have been conducted to confirm the recommendation of this treatment for TKI-induced proteinuria. Isolated proteinuria is not an indication for dose reduction unless nephrotic-range (3.5 g or more per day) or edema, hyperlipidemia, and hypoalbuminemia occur [37]. Acute kidney injury, nephrotic range proteinuria, and TMA are generally considered reasons to discontinue therapy with TKIs [27, 38]. In TMA, anti-VEGF cessation halts further renal deterioration and enables at least partial recovery [39, 40]. Reintroduction of the drug allowing for continued antitumor treatment might be possible at lower doses; however, choosing an appropriate dose for reintroduction may be difficult. In an observational study, continued drug administration or reintroduction resulted in a more severe TMA recurrence in three of four patients requiring a maintenance dose of anti-VEGF agents [41]. Therefore, permanent drug discontinuation should be strongly considered in the case of TMA. It is important, that renal function parameters such as creatinine, blood urea nitrogen, baseline proteinuria, and estimated glomerular filtration rate (eGFR) are measured before initiating TKI treatment. Clinicians are also advised to check for proteinuria and renal function indices before each dose or therapy cycle.

### Patients with CKD or undergoing dialysis

The tyrosine kinase inhibitors are primarily metabolized in the liver by the cytochrome CYP3A4, and their renal excretion does not exceed 23% (Table 2). In population pharmacokinetic analyses, no correlation between TKI exposure and renal function was observed in subjects with mild, moderate, or severe renal impairment who were not on dialysis. These data indicate no need for initial dose adjustment in patients with mild to severe CKD, although caution is advised if creatinine clearance is lower than 30 ml/min/1.73 m2 (Table 2).

**Table 2:** Metabolism of targeted anticancer agents and management indications based on the drugs’ summaries of product characteristics

Drug	Main way of metabolism	Renal excretion of drug and metabolites	Dose reduction required		
			Patients with mild to moderate CKD^a^	Patients with severe CKD^b^	Patients on dialysis
Tyrosine kinase inhibitors					
Sunitinib [125]	Liver, CYP3A4	16%	No	No	No (ND)
Sorafenib [126]	Liver, CYP3A4	19%	No	No	No (ND)
Pazopanib [127]	Liver, CYP3A4	< 4%	No	No (ND)	No (ND)
Axitinib [128]	Liver, CYP3A4	23%	No	No	No (ND)
mTOR inhibitors					
Everolimus [129]	Liver, CYP3A4	5%	No	No (ND)	No (ND)
Temsirolimus [130]	Liver, CYP3A4	4.6%	No (ND)	No (ND)	No (ND)
Immune checkpoint inhibitors					
Ipilimumab [131]	Proteolytic degradation	No	No	No	No (ND)
Nivolumab [132]	Proteolytic degradation	No	No	No	No (ND)
Pembrolizumab [133]	Proteolytic degradation	No	No	No (ND)	No (ND)
Atezolizumab [134]	Proteolytic degradation	No	No	No (ND)	No (ND)

*CKD* chronic kidney disease, *ND* no data

^a^30–90 ml/min/1.73 m2. ^b^< 30 ml/min/1.73 m^2^

Patients treated with TKIs for an extended time may present with declining renal function. Age-related renal dysfunction interferes and prevents determining cause-effect relation between TKI therapy and renal function [42]. A study by Khan et al. [43] reports that in patients treated with sorafenib or sunitinib who developed renal insufficiency during treatment, median serum creatinine clearance declines from 61.5 mL/min to 39.2 mL/min with the median time to > 30% serum creatinine concentration buildup of 4.6 months. In patients who developed renal insufficiency before starting treatment, median creatinine clearance was rather stable (median fall from 38.4 mL/min to 36.2 mL/min) [43]. Renal insufficiency before the start of treatment does not necessarily increase the risk of kidney function deterioration. Still, in some studies, aggravation of pre-existing kidney impairment may comprise 66% of all renal insufficiencies [21]. Kidney injury that develops during treatment is a risk factor for progressive decline in renal function and increases the risk of dose reduction due to renal insufficiency [43].

The pharmacokinetics of sunitinib, sorafenib, and axitinib in patients with mild to severe CKD are similar to those observed in patients with normal renal function [44–46]. The pharmacokinetics of pazopanib in patients with impaired renal function has not been studied. In patients with mRCC, CKD present at baseline or developed during treatment with sunitinib or sorafenib was not associated with unexpected toxicities [43]. Importantly, patients with mRCC and impaired renal function treated with anti-VEGF drugs do not differ in response rate, time to treatment failure, and OS from patients with normal renal function [47]. A retrospective study of 229 patients with mRCC treated with pazopanib demonstrated that renal function at initiation of therapy did not adversely affect pazopanib's safety and efficacy [48]. Therefore, impaired renal function should not prevent the initiation or continuation of anti-angiogenic therapies.

With the development and introduction of targeted therapies for RCC, TKIs were also studied in hemodialyzed (HD) patients. Studies in HD patients demonstrated no clearance of sunitinib, sorafenib, pazopanib, or axitinib from plasma by the dialyzer. The pharmacokinetics of these drugs is rarely influenced by dialysis [49–52]. Therefore, administration of these drugs may take place anytime, regardless of HD timing. Available data from larger studies of mRCC HD patients treated with TKIs are presented in Table 3. TKIs were generally well tolerated, with few reported AEs.

**Table 3:** Summary of larger studies (number of patients ≥ 8) of the use of TKIs in RCC patients with ESRD

Reference	Number of patients	Cancer type	TKI	Dose	Dose reduction required	Response	PFS	OS	Duration of treatment	AEs (Grade ≥ 3)
Josephs et al. [135]	10	RCC	Sunitinib	50 mg/day (3), 37.5 mg/day (5), 25 mg/day (2)	Yes (3)	PR (3), SD (6), PD (1)	NA	NA	NA	Gr 4: HFSR (1); Gr 3: fatigue (3), diarrhea (1), HFSR (1), rash (1)
Kennoki et al. [49]	10	ccRCC (7), papillary RCC (2), unclassified (1)	Sorafenib	200 mg/b.i.d. (1), 200 mg/day to 200 mg/b.i.d. (7)	No	CR (1), PR (3), SD (4), PD (2)	6.3 mo (median)	14.9 mo (median)	11.3 mo (mean)	Gr 5: subarachnoid hemorrhage (1); Gr 4: cerebellar hemorrhage (1); Gr 3: HFSR (1), hypertension (5), diarrhea (4), deceased hemoglobin (6), pneumonitis (1), sepsis (1)
Casper et al. [136]	21	RCC	Sunitinib	25 mg/day (3), 37.5 mg/day (8), 50 mg/day (10)	Yes (5)	CR (1), PR (10), SD (5), PD (2)	15 mo (median)	29 mo (median)	NA	NA
Masini et al. [137]	16	ccRCC (15), unclassified (1)	Sunitinib	50 mg/day (6), 37.5 mg/day (7), 25 mg (2), 12.5 mg (1)	No	PR (7), SD (5)	10.3 mo (median)^a^	22.6 mo (median)^a^	12.7 mo (median)	Gr 3: thrombocytopenia (1)
	8	ccRCC (8)	Sorafenib	400 mg/b.i.d. (4), 400 mg/day (3), 200 mg/day (1)	No	PR (1), SD (7)	10.3 mo (median)^a^	22.6 mo (median)^a^	13.9 mo (median)	Gr 3: nausea (1), fatigue (1), diarrhea (1), symptomatic cardiac ischemia (1)
Shetty et al. [138]	8	ccRCC (5), papillary RCC (3)	Sunitinib	50 mg/day (3), 37.5 mg/day (4), 12.5 mg/day (1)	No	NA	5.6 mo (median)	NA	6.2 mo (median)	None
	7	ccRCC (5), papillary RCC (2)	Sorafenib	400 mg/b.i.d. (2), 600 mg/day (3), 200 mg/b.i.d. (1), 200 mg/day (1)	Yes (2)	NA	4.2 mo (median)	NA	5.3 mo (median)	Gr 3: fatigue (1), HFSR (1)
	9	ccRCC (7), papillary RCC (2)	Pazopanib	800 mg/day (5), 600 mg/day (4)	Yes (6)	NA	NA	NA	11.6 mo (median)	Gr 3: fatigue (2), increased transaminases (1)
Czarnecka et al. [139]	5	ccRCC	Sunitinib	50 mg/day (4), 37.5 mg/day (1)	Yes (2)	SD (4), PD (1)	8 mo (median)^b^	NA	NA	None
	3	ccRCC	Sorafenib	400 mg/b.i.d	No	PR (1), SD (2)	8 mo (median)^b^	NA	NA	Gr 4: hypertension (1)
	1	ccRCC	Pazopanib	400 mg/b.i.d	No	PD (1)	8 mo (median)^b^	NA	NA	None
Omae et al. [140]	20	ccRCC (8), papillary RCC (8)	Sorafenib	200 mg/day (5), 400 mg/day (10), 600 mg/day (5)	No^c^	CR (1), PR (2), SD (11), PD (3)	6.3 mo (median)	14.2 mo (median)	4.7 mo (median)	Gr 5: LVDD (1), subarachnoid hemorrhage (1); Gr 4: cerebral hemorrhage (1); Gr 3: HSFR (1), hypertension (3), LVDD (1), fatigue (2), anorexia (2), diarrhea (3), syncope (1), pneumonitis (1), sepsis (1), anemia (5)
Ishihara et al. [57]	8	ccRCC (4), papillary RCC (2), ACD–RCC (1), MiT family translocation RCC (1)	Axitinib	14 mg/day (2), 10 mg/day (4), 8 mg/day (2)^d^	Yes (3)	PR (1), SD (5), PD (2)	8.1 mo (median)	13.3 mo (median)	NA	None

*ACD–RCC* acquired cystic disease‐associated renal cell carcinoma, *AEs* adverse reactions, *b.i.d.* twice a day, *ccRCC* clear cell renal cell carcinoma, *CR* complete response, *ESRD* end-stage renal disease, *Gr* grade, *HFSR* hand-foot skin reaction, *LVDD* left ventricular diastolic dysfunction, *mo* months, *NA* not available, *OS* overall survival, *PD* progressive disease, *PFS* progression free survival, *PR* partial response, *RCC *renal cell carcinoma, *SD* stable disease, *TKI* tyrosine kinase inhibitor

^a^Estimated for the entire cohort of patients treated with sunitinib and sorafenib

^b^Estimated for the entire cohort of patients treated with sunitinib, sorafenib and pazopanib

^c^Four patients discontinued therapy because of serious AEs

^d^Maximum daily dose

Nevertheless, a systematic review by Leonetti et al. [53] showed a higher incidence of sorafenib-related AEs in mRCC dialysis patients compared to the general mRCC population. Incidents of severe AEs such as subarachnoid hemorrhage resulting in the patient's death were also noted (Table 3). The median PFS of 10.9 and 9 months and OS of 18.4 and 12.5 months presented in expanded-access trials of sunitinib and sorafenib, respectively [54, 55], are comparable to those reported in HD patients (Table 3). Evidence from a small number of cases also suggests a good efficacy and tolerability of pazopanib and axitinib treatment in mRCC patients undergoing dialysis [56, 57]. At this time, there are no established evidence-based guidelines on the management of such patients. Based on the available literature, the initial dose adjustment of TKIs seems unnecessary in patients receiving dialysis (Table 2). Long-term HD patients have a higher risk of cardiovascular comorbidities and should be monitored closely, as cardiovascular events are likely to be more frequent in this population [49]. Therapy administration should be performed with caution, followed by increased surveillance of AEs and appropriate dose reduction if AEs occur. Most importantly, dialysis should not be regarded as a contraindication to this type of treatment.

## Renal toxicities of mTORis therapy

### Incidence

Inhibitors of mammalian target of rapamycin (mTORis) such as temsirolimus and everolimus are well-established options for treating mRCC in further lines after TKI failure. Commonly reported side effects with mTORis include stomatitis, rash, fatigue, asthenia, diarrhea, metabolic complications, infections, and noninfectious pneumonitis [58]. Adverse renal effects, such as AKI, have also been reported. In a meta-analysis of nine randomized clinical trials assessing renal toxicity of mTORis, all-grade AKI and high-grade AKI occurred in 15.7% and 4.2% of patients, respectively. Interestingly, the AKI incidence rates did not differ significantly between mTORis and other drugs tested in RCC randomized clinical trials [59]. In another meta-analysis, AKI was the second most common cause of fatal AEs in patients receiving mTORis for cancer, representing 5.7% of all study deaths [60]. The incidence of proteinuria in mRCC patients treated with mTORis has not been reported in the literature; however, it has been described in renal transplant recipients receiving everolimus [61].

### Mechanisms of nephrotoxicity

The mTOR complex plays an important role in the process of kidney regeneration and recovery. Although mTOR activity is low or absent in the healthy kidney, it increases markedly after ischemia–reperfusion injury [62]. Inhibition of mTOR by rapamycin delayed renal recovery and repair after AKI in animal models but, importantly, renal function fully recovered after several days, despite continued treatment [63]. Everolimus was also shown to have antiproliferative effects and, through induction of autophagy, to aggravate tubular dysfunction during recovery from kidney injury in a rat model [64]. Biopsy-proven acute tubular necrosis after starting mTORis therapy was reported in a case series of four patients [65]. The effect of everolimus on tubular cells can be reflected by an accumulation of the cellular protein LC3 A in the urine, which might in the future prove to be a useful marker of AKI induced by mTOR inhibition [64].

Moreover, everolimus induced renal function deterioration and proteinuria by inhibiting the proliferative activity associated with reduced VEGF expression in a remnant kidney model. This mechanism may be particularly important in patients with RCC after nephrectomy [66]. Another potential mechanism for mTORis nephrotoxicity is based on the fact that rapamycin inhibits mTORC2, a multiprotein complex containing mTOR, which activates the Akt/PKB kinase [67]. Thus, mTORis inhibit the Akt pathway, which is essential for maintaining cell survival and signaling. It is still unclear whether mTORis cause renal dysfunction directly or through impairment of kidney repair in response to stress caused by other nephrotoxic factors [65, 66].

### Management

ACEIs and ARBs are indicated in the treatment of mTORi-associated proteinuria [68]. Caution should be taken while administering ACEIs in combination with temsirolimus, as sporadic cases of angioneurotic edema have been reported in the drug’s summary of product characteristics. If grade 3 renal toxic effects develop during mTORi therapy, treatment should be suspended and resumed upon renal function recovery. In AKI or grade 4 proteinuria, permanent discontinuation of treatment is generally recommended [38]. In a Korean retrospective study, the occurrence of AKI in RCC patients did not require everolimus discontinuation in 9 of 14 cases. The authors suggested that the treatment decision should be made via a multidisciplinary approach, including the assessment of oncological benefits of everolimus and other therapeutic options [69].

### Patients with CKD or undergoing dialysis

mTORis such as temsirolimus and everolimus are metabolized mostly by the liver. In population pharmacokinetic analyses, creatinine clearance was not affected by the clearance of everolimus in patients with mild to moderate CKD. No clinical studies were conducted with temsirolimus in patients with decreased renal function (Table 2). However, considering their very small renal excretion, no dosage adjustment of everolimus or temsirolimus is recommended in patients with renal impairment.

RCC patients with impaired renal function are at a higher risk of developing AKI with mTORis. In the previously mentioned Korean study [69], AKI incidence in patients receiving everolimus increased as the baseline eGFR decreased. Moreover, baseline eGFR was an independent risk factor for the development of everolimus-associated AKI [69]. In a retrospective analysis of 18 patients with non-dialysis dependent CKD and mRCC treated with mTORis, elevated creatinine level was noted in 77% of patients. The efficacy and safety of mTORis use were similar to patients with normal renal function [70].

mTORis do not require dose adjustment in hemodialyzed patients, as their blood concentration is not altered by dialysis [71, 72]. Table 4 summarizes available evidence of mTORis use in RCC patients on hemodialysis. Grade 3 AEs occurred in ten cases, and no grade 4 AEs were observed. The estimated median PFS and OS of 9.0 and 15.7 months, respectively, reported by Guida et al. in RCC patients on dialysis receiving everolimus [73], are in line with those reported in the phase III everolimus RECORD-1 study [74]. Therefore, mTORis seem to be an effective and safe treatment option for patients with mRCC and severe renal impairment requiring dialysis. Therefore, the use of mTORis should not be contraindicated in this subset of patients.

**Table 4:** Summary of published cases of the use of mTORis in RCC patients with ESRD

Reference	Number of patients	Cancer type	mTORi	Dose	Dose reduction required	Response	PFS	OS	Duration of treatment	AEs (Grade ≥ 3)
Thiery-Vuillemin et al. [72]	2	RCC	Everolimus	5 mg p.o./day^a^	Yes (1)^a^	NA	NA	NA	NA	Gr 3: hyperglycemia (1), asthenia (1)
Syrios et al. [141]	2	ccRCC, chromophobe RCC	Everolimus	10 mg p.o./day	No	NA	NA	NA	NA	None
Miyake et al. [142]	10	ccRCC (7), papillary RCC (3)	Temsirolimus	25 mg i.v./week	Yes (4)	SD (9), PD (1)	NA	NA	13 mo (median)	Gr 3: asthenia (1), anemia (1), thrombocytopenia (2)
Shetty et al. [138]	3	ccRCC (1), papillary RCC (2)	Temsirolimus	25 mg i.v./week	No	NA	NA	NA	1.9 mo (median)	None
	7	cRCC (4), papillary RCC (3)	Everolimus	10 mg p.o./day	Yes (1)	NA	NA	NA	4.7 mo (median)	One patient discontinued everolimus because of pneumonitis
Guida et al. [73]	11	ccRCC (11)	Everolimus	10 mg p.o./day (10); 5 mg p.o./day (1)^b^	No	PR (1), SD (7)	9 mo (median)	15.7 mo (median)	14.4 (mean)	Gr 3: cutaneous rash (1), anemia (1), thrombocytopenia (1), dyspnea (1)
Omae et al. [143]	4	ccRCC (2), papillary RCC (2)	Everolimus	5 mg p.o./day (3); 10 mg p.o./day (1)^c^	No	SD (4)	NA	NA	6.7mo (median)	None
	2	ccRCC (1), papillary RCC (1)	Temsirolimus	25 mg i.v./week (1), 20 mg i.v./week (1)^c^	No	SD (2)	NA	NA	9.5mo (median)	None

*AEs* adverse reactions, *ccRCC* clear cell renal cell carcinoma, *ESRD* end-stage renal disease, *Gr* grade, *i.v.* intravenous, *mo* months, *NA* not available, *OS* overall survival, *PD *progressive disease, *PFS* progression free survival, *p.o.* per os, *PR* partial response, *RCC* renal cell carcinoma, *SD* stable disease, *mTORi* mammalian target of rapamycin inhibitor

^a^Everolimus starting dose was 5 mg/day with the possibility of escalation according to the tolerance after first pharmacokinetic assessment. One patient experienced escalation to 10 mg/day, but required dose reduction to 5 mg/day due to adverse events

^b^Reduced dose was started in a patient receiving peritoneal dialysis due to individual physician’s choice

^c^Therapy initiated at a low dose and titrated up to a maintenance dose based on tumor response and AEs

## Renal toxicities of immune checkpoint inhibitors

### Incidence

In the last years, ICPIs have become the standard first line of treatment in mRCC [75]. Among the drugs currently approved are cytotoxic T lymphocyte-associated protein 4 (CTLA-4) blocking antibodies (ipilimumab), programmed cell death protein 1 (PD-1) blocking antibodies (nivolumab, pembrolizumab), and PD-ligand 1 antibodies (atezolizumab). Several organ systems are affected by the use of ICPIs, including the central nervous system and cardiovascular, respiratory, musculoskeletal, and hematologic systems. However, the gastrointestinal tract, endocrine glands, skin, and liver seem to be the most commonly involved [76].

Adverse renal effects of ICPIs are rare but increasingly described. They include AKI, proteinuria, and electrolyte abnormalities. The frequency of immune checkpoint inhibitor-associated AKI (ICPI-AKI) does not seem to differ significantly between CTLA-4 and PD1 targeting drugs [77]. A recent review of 48 clinical trials involving 11,482 patients treated with PD-1 inhibitors reported a pooled relative risk for AKI of 4.19 (95% confidence interval, 1.57–11.18) when compared with non-nephrotoxic controls and an estimated incidence of 2.2% [78]. These numbers are likely underestimated, as they reflect data obtained in clinical trials, which might not be applicable in a standard clinical setting.

### Combination therapy

In recent years, clinical investigations have focused on two types of combination regimens: combinations of ICPIs and combinations of ICPIs and TKIs. In April 2018, the FDA approved the combination therapy of ipilimumab and nivolumab to treat intermediate or poor-risk advanced RCC. In the CheckMate 214 study evaluating the combination of nivolumab plus ipilimumab as first-line treatment for advanced RCC, increased blood creatinine was present in 7.3% of patients. AKI occurred in 2.2% of patients treated with combination therapy [79]. In an analysis of data from phase II and III clinical trials, the incidence of ICPI-AKI was estimated to be higher in patients receiving combination ipilimumab/nivolumab therapy (4.9%) compared to monotherapy (pembrolizumab, 1.4%, nivolumab, 1.9%, ipilimumab, 2%) [80]. Recently, positive results of studies assessing outcomes of ICPIs and TKIs combinations led to FDA approvals of pembrolizumab plus axitinib and avelumab plus axitinib in first-line treatment of advanced RCC. In a phase III JAVELIN Renal 101 study investigating avelumab plus axitinib for advanced RCC, no renal AEs were reported with the data cutoff of ≥ 10% for any grade events and ≥ 5% for grade ≥ 3 events [81]. In an extended follow-up from a phase III study of pembrolizumab plus axitinib in RCC treatment (KEYNOTE-426), AKI was present in 5 of 429 patients (1.2%) and nephritis in 8 patients (1.9%) [82]. Importantly, in both studies, combination therapy was not associated with a higher incidence of AEs than sunitinib alone.

Additionally, in a phase I study of mRCC patients treated with nivolumab combined with sunitinib or pazopanib (CheckMate 016 study), treatment was associated with increased blood creatinine in 33.3% and 5%, respectively. AKI leading to treatment discontinuation occurred in 9.1% of patients in the study's nivolumab plus sunitinib arm [83]. Glomerulonephritis was also reported in a series of three patients treated with immunotherapy and TKIs [84]. Combinations of ICPIs and TKIs are currently tested in various clinical trials; they offer promising results regarding improved clinical outcomes while maintaining tolerable side effect profiles resulting in wider use in the nearest future. Currently available data do not suggest that combination therapy is associated with previously unknown toxicities. Considering these data and renal AEs observed in monotherapy, special caution and close monitoring of renal function might be necessary to prevent and early detect possible toxicities from combination therapy.

### Mechanisms of nephrotoxicity

Acute tubulointerstitial nephritis (ATIN), either alone or other kidney lesions such as acute tubular injury or glomerular disease, is the most common histopathologic finding ICPI–AKI on kidney biopsy [85]. In the largest study of patients with ICPI–AKI up to date, ATIN was the dominant lesion in 93% of the 60 patients biopsied [86]. In a study by Mamlouk et al., ATIN was present in 14 of 16 cases; however, nine of these cases were associated with glomerular disease, including pauci-immune glomerulonephritis, IgA nephropathy, and other pathologies [87].

The precise mechanisms of ICPI–AKI are not yet known, with two hypotheses suggested by most researchers [80, 88–90]. Through the inhibition of specific receptors (e.g., CTLA-4, PD-1) or their ligands (PD-L1), ICPIs “release the brakes” of the immune system, allowing T-cells to become activated and exert antitumor activity [91]. Mice with knockout CTLA-4 or PD-1 genes have been shown to develop autoimmunity against specific organs, including glomerulonephritis, driven by the emergence of antigen-specific T-cells targeting self-antigens [92, 93]. Blockade of PD-1 or CTLA-4 with ICPIs in humans may lead to a loss of tolerance against endogenous kidney antigens and cause cytotoxic injury to the kidney. The exact antigen has not yet been identified but is possibly expressed by tubular cells based on ATIN's dominant finding on biopsy. Alternatively, it is also possible that ICPIs lead to activation of memory T-cells previously primed by other haptens causing ATIN, such as proton pump inhibitors (PPIs) or nonsteroidal anti-inflammatory drugs (NSAIDs). In support of this theory, in a recent large multicentre study, nearly 70% of patients with ICPI–AKI received a potential ATIN-causing medication, including PPIs in over 50% of cases. Concomitant use of PPIs was also an independent risk factor for the development of ICPI–AKI. [86].

### Management

The European Society for Medical Oncology (ESMO) guidelines recommend that serum sodium, potassium, creatinine, and urea should be measured before every infusion of ICPIs. Baseline urinalysis, with quantification of proteinuria or microalbuminuria if present, before initiation of ICPI therapy is also advised [94]. Baseline assessment and monitoring of the number of leukocytes in urinalysis could also be helpful, since sterile pyuria and/or leukocyte casts may suggest an inflammatory kidney lesion, although they are not specific for ATIN diagnosis [95]. If renal dysfunction develops during treatment, alternative AKI etiologies, such as hypovolemia, infection, contrast-enhanced nephropathy, and urinary tract obstruction, should be initially ruled out. Immunotherapy should be temporarily discontinued until further clarification of the cause of AKI. The role of kidney biopsy in patients who developed AKI while undergoing ICPI treatment is debated. American Society of Clinical Oncology (ASCO) and ESMO guidelines recommend proceeding directly with immunosuppressive therapy without a kidney biopsy unless an alternative cause of AKI is suspected [96, 97].

Once the diagnosis of ICPI–AKI is made, the discontinuation of ICPI therapy and glucocorticoid administration is generally recommended. Table 5 summarizes the published guidelines for the management of ICPI-related nephritis [96, 97]. In a multicenter study of 138 patients, this approach was associated with partial or complete kidney function recovery in 95% of cases [86]. Considering the potential influence of concomitant medications in the pathogenesis of ICPI–AKI, drugs that are known to induce ATIN, such as PPIs or NSAIDs, should be recognized and discontinued if possible [98]. Most authors also suggest that patients developing initial ICPI–AKI episode can be safely rechallenged with ICPIs once kidney function improves and corticosteroid administration is complete or nearly complete. Observational data support that statement. Out of 31 patients rechallenged with an ICPI after the initial episode of ICPI–AKI only 7 (23%) experienced recurrent AKI, 6 of whom had complete or partial renal recovery [86].

**Table 5:** Grading of ICPI-related nephritis and management by severity

Grade^a^	Management
G1: creatinine > ULN–1.5 × ULN	Continue ICPI Discontinue nephrotoxic drugs Monitor renal function and proteinuria Exclude alternative causes (dehydration, recent i.v. contrast, UTI, medications, obstruction, hypotension, hypertension)
G2: creatinine > 1.5–3.0 × baseline; > 1.5–3.0 × ULN	Withhold ICPI Discontinue nephrotoxic drugs Ensure hydration Monitor renal function and proteinuria Consult nephrologist Consider biopsy Exclude alternative causes (as above) Initiate steroids (0.5–1 mg/kg/day oral prednisolone or equivalent)
G3: creatinine > 3.0 × baseline; > 3.0–6.0 × ULN	Admit patient for monitoring and fluid balance Withhold ICPI, consider permanent discontinuation Discontinue nephrotoxic drugs Monitor renal function and proteinuria Consult nephrologist Consider biopsy Evaluate alternative causes (as above) Initiate steroids (1–2 mg/kg/day i.v. prednisolone or equivalent)
G4: creatinine > 6.0 × ULN	As grade 3 Treat in a center where renal replacement therapy is available

*ICPI* immune checkpoint inhibitor, *i.v.* intravenous, *ULN* upper limit of normal, *UTI* urinary tract infection

^a^According to CTCAE v 5.0

### Patients with CKD or undergoing dialysis

Underlying kidney disease may cause significant difficulties in the treatment of RCC patients with ICPIs. Most clinical trials do not include patients with moderate to severe kidney failure. Thus, ICPIs have not been widely studied in patients with renal failure or end-stage renal disease on dialysis. Because ICPIs undergo proteolytic degradation and not renal excretion, the lower glomerular filtration rate is not expected to impact their pharmacokinetics. In fact, in the case of nivolumab, the pharmacokinetics is linear in the dose range of 0.1 to 10 mg/kg. There is no relationship between renal function status and pharmacokinetics [99]. In the IMvigor210 phase II trial of atezolizumab for cisplatin-ineligible urothelial carcinoma, 70% of patients had eGFR between 30 and 60 ml/min, a comparable response rate and no loss in median eGFR was reported [100].

Considering the large molecular weights of ICPIs, drug filtration through the renal glomeruli or dialysis pores is unlikely. There are currently few published data on the use of ICPIs in patients with ESRD on hemodialysis (Table 6). Apart from nivolumab, other ICPIs have not been studied in this group of patients. Of the 20 described cases, in 80% of patients the treatment resulted in partial response or stable disease. Four grade 3 immune-related adverse events (irAEs) were observed, and no grade 4 irAEs have been reported up to date. Despite the small sample size, Tachibana et al. revealed no significant differences between the ESRD and non-ESRD groups in terms of PFS and OS [101]. The presented evidence suggests that renal impairment or ESRD may not be a contraindication for ICPI use in RCC patients.

**Table 6:** Summary of published cases of the use of immunotherapy in RCC patients with ESRD

Reference	Number of patients	Cancer type	ICPI	Dose	Dose reduction required	Response	PFS	OS	Duration of treatment	irAEs (Grade ≥ 3)
Carlo et al. [144]	1	ccRCC	Nivolumab	NA	No	PR	> 8 mo	NA	> 8 mo	None
Tabei et al. [145]	1	ccRCC	Nivolumab	3 mg/kg every 2 wk	No	PR	> 6 mo	NA	> 6 mo	None
Ansari et al. [146]	1	ccRCC	Nivolumab	3 mg/kg every 2 wk ➔ 240 mg every 2 wk	No	PR	> 22 mo	NA	> 22 mo	None
Cheun et al. [147]	2	RCC (1), ccRCC (1)	Nivolumab	100 mg every 3 wk, 3 mg/kg every 3 wk	No	PR (1), SD (1)	6.8 mo (1), > 9 mo (1)	15 mo (1), > 9mo (1)	NA	None
Tachibana et al. [101]	7	RCC (2), ccRCC (5)	Nivolumab	3 mg/kg every 2 wk	No	PR (1), SD (4), PD (1), NA (1)	5.9 mo (median)	11.9 mo (median)	6 mo (median)	Gr 3: fatigue (1)
Vitale et al. [148]	8	ccRCC	Nivolumab	3 mg/kg every 2 wk ➔ 240 mg every 2 wk or 480 mg every 4 wk	No	PR (1), SD (5), PD (2)	16 mo (median)	26 mo (median)	9 mo (median)	Gr 3: diarrhea (1), asthenia (1), anorexia (1)

*ccRCC* clear cell renal cell carcinoma, *Gr* grade, *ESRD* end-stage renal disease, *ICPI* immune checkpoint inhibitor, *irAEs* immune-related adverse reactions, *mo* months, *NA* not available, *OS* overall survival, *PD* progressive disease, *PFS* progression free survival, *PR* partial response, *RCC* renal cell carcinoma, *SD* stable disease, *wk* weeks

## Conclusions

Renal toxicity of targeted anticancer therapies represents an increasingly recognized problem for clinicians involved in treating patients with mRCC. As these AEs can lead to dose reductions or interruption of treatment, which might have a negative effect on the patient’s survival, correct recognition and management of specific toxic effects are especially important.

There are several reasons why the relationships between targeted agents and the kidneys remain largely unexplored. Large randomized, controlled, phase III trials do not enroll patients with reduced kidney function; renal AEs are often not reported; the methodology and terminology differ across oncological trials (for example, definitions of creatinine elevation, CKD, or AKI); and most reports of patients on hemodialysis involve only single cases or small case series. Furthermore, direct comparison of PFS and OS is often not possible since reported data include the usage of drugs in various lines of therapy, differing starting doses, and subsequent dose escalation or reduction schemes.

Careful design of clinical trials focusing on renal toxic effects should shed more light on a group of patients for whom targeted therapies are a viable first- and second-line option. Further studies are needed to establish treatment guidelines for patients with impaired renal function and/or treated with chronic dialysis.

Because of the complex relationship between cancer, kidneys, and novel therapeutic agents, close collaboration between clinical oncologists, urologists, and nephrologists is crucial. The emergence of effective targeted therapies for mRCC significantly improved patients’ prognoses, at the cost of a whole new spectrum of renal adverse events that differ from those observed with conventional cytotoxic chemotherapy. Therefore, nephrologists should become acquainted with various aspects of cancer care, including the biology of RCC and molecular mechanisms of action of novel anticancer drugs. Onconephrology is an evolving and expanding subspecialty that relies on a multidisciplinary approach necessary to provide cutting-edge care for RCC patients with kidney impairment [102, 103].

This analysis highlights that patients with mRCC, including those on hemodialysis, generally benefit from targeted treatment in PFS and OS. Renal toxicities of targeted therapies differ in incidence but are generally mild to moderate in severity and can be managed effectively. The occurrence of AEs should not necessarily result in treatment discontinuation, and even if that decision is made, treatment can be resumed in specific situations. That said, therapy selection, administration, and toxicity management in mRCC patients undergoing dialysis should be performed with caution and increased monitoring of AEs. Close cooperation between oncologists and nephrologists in managing renal toxic effects should be encouraged to improve patients' outcome.

## References

[1] Siegel RL, Miller KD (2020). Jemal A (2020) Cancer statistics. CA Cancer J Clin.

[2] Macleod LC, Hotaling JM, Wright JL, Davenport MT, Gore JL, Harper J, White E (2013). Risk factors for renal cell carcinoma in the VITAL study. J Urol.

[3] Capitanio U, Bensalah K, Bex A, Boorjian SA, Bray F, Coleman J, Gore JL, Sun M, Wood C, Russo P (2019). Epidemiology of renal cell carcinoma. Eur Urol.

[4] Joh HK, Willett WC, Cho E (2011). Type 2 diabetes and the risk of renal cell cancer in women. Diabetes Care.

[5] Huang WC, Levey AS, Serio AM, Snyder M, Vickers AJ, Raj GV, Scardino PT, Russo P (2006). Chronic kidney disease after nephrectomy in patients with renal cortical tumours: a retrospective cohort study. Lancet Oncol.

[6] Kompotiatis P, Thongprayoon C, Manohar S, Cheungpasitporn W, Gonzalez Suarez ML, Craici IM, Mao MA, Herrmann SM (2019). Association between urologic malignancies and end-stage renal disease: a meta-analysis. Nephrology (Carlton).

[7] Chewcharat A, Thongprayoon C, Bathini T, Aeddula NR, Boonpheng B, Kaewput W, Watthanasuntorn K, Lertjitbanjong P, Sharma K, Torres-Ortiz A, Leeaphorn N, Mao MA, Khoury NJ, Cheungpasitporn W (2019). Incidence and mortality of renal cell carcinoma after kidney transplantation: a meta-analysis. J Clin Med.

[8] Dy GW, Gore JL, Forouzanfar MH, Naghavi M, Fitzmaurice C (2017). Global burden of urologic cancers, 1990–2013. Eur Urol.

[9] Neuzillet Y, Tillou X, Mathieu R, Long JA, Gigante M, Paparel P, Poissonnier L, Baumert H, Escudier B, Lang H, Rioux-Leclercq N, Bigot P, Bernhard JC, Albiges L, Bastien L, Petit J, Saint F, Bruyere F, Boutin JM, Brichart N, Karam G, Branchereau J, Ferriere JM, Wallerand H, Barbet S, Elkentaoui H, Hubert J, Feuillu B, Theveniaud PE, Villers A, Zini L, Descazeaux A, Roupret M, Barrou B, Fehri K, Lebret T, Tostain J, Terrier JE, Terrier N, Martin L, Dugardin F, Galliot I, Staerman F, Azemar MD, Irani J, Tisserand B, Timsit MO, Sallusto F, Rischmann P, Guy L, Valeri A, Deruelle C, Azzouzi AR, Chautard D, Mejean A, Salomon L, Rigaud J, Pfister C, Soulie M, Kleinclauss F, Badet L, Patard JJ, Comite de Transplantation de l'Association Francaise dU, Comite de Cancerologie de l'Association Francaise dU (2011). Renal cell carcinoma (RCC) in patients with end-stage renal disease exhibits many favourable clinical, pathologic, and outcome features compared with RCC in the general population. Eur Urol.

[10] Chen K, Huang HH, Aydin H, Tan YH, Lau WK, Cheng CW, Yuen JS (2015). Renal cell carcinoma in patients with end-stage renal disease is associated with more favourable histological features and prognosis. Scand J Urol.

[11] Tsuzuki T, Iwata H, Murase Y, Takahara T, Ohashi A (2018). Renal tumors in end-stage renal disease: a comprehensive review. Int J Urol.

[12] Kim CS, Bae EH, Ma SK, Kweon SS, Kim SW (2014). Impact of partial nephrectomy on kidney function in patients with renal cell carcinoma. BMC Nephrol.

[13] Schmid M, Krishna N, Ravi P, Meyer CP, Becker A, Dalela D, Sood A, Chun FK, Kibel AS, Menon M, Fisch M, Trinh QD, Sun M (2016) Trends of acute kidney injury after radical or partial nephrectomy for renal cell carcinoma. Urol Oncol 34 (7):293 e291–293–e210. 10.1016/j.urolonc.2016.02.018 10.1016/j.urolonc.2016.02.018 27033047

[14] Grimaldi G, Reuter V, Russo P (1998). Bilateral non-familial renal cell carcinoma. Ann Surg Oncol.

[15] Janzen NK, Kim HL, Figlin RA, Belldegrun AS (2003). Surveillance after radical or partial nephrectomy for localized renal cell carcinoma and management of recurrent disease. Urol Clin N Am.

[16] Cosmai L, Porta C, Perazella MA, Launay-Vacher V, Rosner MH, Jhaveri KD, Floris M, Pani A, Teuma C, Szczylik CA, Gallieni M (2018). Opening an onconephrology clinic: recommendations and basic requirements. Nephrol Dial Transplant.

[17] Motzer RJ, Hutson TE, Tomczak P, Michaelson MD, Bukowski RM, Rixe O, Oudard S, Negrier S, Szczylik C, Kim ST, Chen I, Bycott PW, Baum CM, Figlin RA (2007). Sunitinib versus interferon alfa in metastatic renal-cell carcinoma. N Engl J Med.

[18] Motzer RJ, Hutson TE, Tomczak P, Michaelson MD, Bukowski RM, Oudard S, Negrier S, Szczylik C, Pili R, Bjarnason GA, Garcia-del-Muro X, Sosman JA, Solska E, Wilding G, Thompson JA, Kim ST, Chen I, Huang X, Figlin RA (2009). Overall survival and updated results for sunitinib compared with interferon alfa in patients with metastatic renal cell carcinoma. J Clin Oncol.

[19] Zhu X, Stergiopoulos K, Wu S (2009). Risk of hypertension and renal dysfunction with an angiogenesis inhibitor sunitinib: systematic review and meta-analysis. Acta Oncol.

[20] Czarnecka AM, Sobczuk P, Korniluk J, Spychalska M, Bogusz K, Owczarek A, Brodziak A, Labochka D, Moszczuk B, Szczylik C (2017). Long-term response to sunitinib: everolimus treatment in metastatic clear cell renal cell carcinoma. Future Oncol.

[21] Baek SH, Kim H, Lee J, Kim DK, Oh KH, Kim YS, Han JS, Kim TM, Lee SH, Joo KW (2014). Renal adverse effects of sunitinib and its clinical significance: a single-center experience in Korea. Korean J Intern Med.

[22] Gupta S, Parsa V, Heilbrun LK, Smith DW, Dickow B, Heath E, Vaishampayan U (2011). Safety and efficacy of molecularly targeted agents in patients with metastatic kidney cancer with renal dysfunction. Anticancer Drugs.

[23] Rini BI, Escudier B, Tomczak P, Kaprin A, Szczylik C, Hutson TE, Michaelson MD, Gorbunova VA, Gore ME, Rusakov IG, Negrier S, Ou YC, Castellano D, Lim HY, Uemura H, Tarazi J, Cella D, Chen C, Rosbrook B, Kim S, Motzer RJ (2011). Comparative effectiveness of axitinib versus sorafenib in advanced renal cell carcinoma (AXIS): a randomised phase 3 trial. Lancet.

[24] Motzer RJ, Hutson TE, Cella D, Reeves J, Hawkins R, Guo J, Nathan P, Staehler M, de Souza P, Merchan JR, Boleti E, Fife K, Jin J, Jones R, Uemura H, De Giorgi U, Harmenberg U, Wang J, Sternberg CN, Deen K, McCann L, Hackshaw MD, Crescenzo R, Pandite LN, Choueiri TK (2013). Pazopanib versus sunitinib in metastatic renal-cell carcinoma. N Engl J Med.

[25] Miyake H, Muramaki M, Imai S, Harada K, Fujisawa M (2016). Changes in renal function of patients with metastatic renal cell carcinoma during treatment with molecular-targeted agents. Target Oncol.

[26] Zhang W, Feng LJ, Teng F, Li YH, Zhang X, Ran YG (2020). Incidence and risk of proteinuria associated with newly approved vascular endothelial growth factor receptor tyrosine kinase inhibitors in cancer patients: an up-to-date meta-analysis of randomized controlled trials. Expert Rev Clin Pharmacol.

[27] Izzedine H, Massard C, Spano JP, Goldwasser F, Khayat D, Soria JC (2010). VEGF signalling inhibition-induced proteinuria: mechanisms, significance and management. Eur J Cancer.

[28] Usui J, Glezerman IG, Salvatore SP, Chandran CB, Flombaum CD, Seshan SV (2014). Clinicopathological spectrum of kidney diseases in cancer patients treated with vascular endothelial growth factor inhibitors: a report of 5 cases and review of literature. Hum Pathol.

[29] Stylianou K, Lioudaki E, Papadimitraki E, Kokologiannakis G, Kroustalakis N, Liotsi C, Giannakakis K, Georgoulias V, Daphnis E (2011). Crescentic glomerulonephritis associated with vascular endothelial growth factor (VEGF) inhibitor and bisphosphonate administration. Nephrol Dial Transplant.

[30] Rolleman EJ, Weening J, Betjes MGH (2009). Acute nephritic syndrome after anti-VEGF therapy for renal cell carcinoma. Nephrol Dial Transplant.

[31] Schwandt A, Wood LS, Rini B, Dreicer R (2009). Management of side effects associated with sunitinib therapy for patients with renal cell carcinoma. Onco Targets Ther.

[32] Kappers MH, van Esch JH, Sluiter W, Sleijfer S, Danser AH, van den Meiracker AH (2010). Hypertension induced by the tyrosine kinase inhibitor sunitinib is associated with increased circulating endothelin-1 levels. Hypertension.

[33] Schrijvers BF, Flyvbjerg A, De Vriese AS (2004). The role of vascular endothelial growth factor (VEGF) in renal pathophysiology. Kidney Int.

[34] Simons M, Schwarz K, Kriz W, Miettinen A, Reiser J, Mundel P, Holthofer H (2001). Involvement of lipid rafts in nephrin phosphorylation and organization of the glomerular slit diaphragm. Am J Pathol.

[35] Liu Y, Zhou L, Chen Y, Liao B, Ye D, Wang K, Li H (2019). Hypertension as a prognostic factor in metastatic renal cell carcinoma treated with tyrosine kinase inhibitors: a systematic review and meta-analysis. BMC Urol.

[36] Sorich MJ, Rowland A, Kichenadasse G, Woodman RJ, Mangoni AA (2016). Risk factors of proteinuria in renal cell carcinoma patients treated with VEGF inhibitors: a secondary analysis of pooled clinical trial data. Br J Cancer.

[37] Alasker A, Meskawi M, Sun M, Ismail S, Hanna N, Hansen J, Tian Z, Bianchi M, Perrotte P, Karakiewicz PI (2013). A contemporary update on rates and management of toxicities of targeted therapies for metastatic renal cell carcinoma. Cancer Treat Rev.

[38] Porta C, Cosmai L, Gallieni M, Pedrazzoli P, Malberti F (2015). Renal effects of targeted anticancer therapies. Nat Rev Nephrol.

[39] Eremina V, Jefferson JA, Kowalewska J, Hochster H, Haas M, Weisstuch J, Richardson C, Kopp JB, Kabir MG, Backx PH, Gerber HP, Ferrara N, Barisoni L, Alpers CE, Quaggin SE (2008). VEGF inhibition and renal thrombotic microangiopathy. N Engl J Med.

[40] Bollee G, Patey N, Cazajous G, Robert C, Goujon JM, Fakhouri F, Bruneval P, Noel LH, Knebelmann B (2009). Thrombotic microangiopathy secondary to VEGF pathway inhibition by sunitinib. Nephrol Dial Transpl.

[41] Izzedine H, Escudier B, Lhomme C, Pautier P, Rouvier P, Gueutin V, Baumelou A, Derosa L, Bahleda R, Hollebecque A, Sahali D, Soria JC (2014). Kidney diseases associated with anti-vascular endothelial growth factor (VEGF): an 8-year observational study at a single center. Medicine (Baltimore).

[42] Launay-Vacher V, Ayllon J, Janus N, Medioni J, Deray G, Isnard-Bagnis C, Oudard S (2011). Evolution of renal function in patients treated with antiangiogenics after nephrectomy for renal cell carcinoma. Urol Oncol.

[43] Khan G, Golshayan A, Elson P, Wood L, Garcia J, Bukowski R, Rini B (2010). Sunitinib and sorafenib in metastatic renal cell carcinoma patients with renal insufficiency. Ann Oncol.

[44] Khosravan R, Toh M, Garrett M, La Fargue J, Ni G, Marbury TC, Swan SK, Lunde NM, Bello CL (2010). Pharmacokinetics and safety of sunitinib malate in subjects with impaired renal function. J Clin Pharmacol.

[45] Smith W, Marbury T, Kipnes M, Cihon F, Lettieri J, Mazzu A (2007). Effects of renal impairment on the pharmacokinetics of sorafenib and its metabolites. Cancer Res.

[46] Chen Y, Rini BI, Motzer RJ, Dutcher JP, Rixe O, Wilding G, Stadler WM, Tarazi J, Garrett M, Pithavala YK (2016). Effect of renal impairment on the pharmacokinetics and safety of axitinib. Target Oncol.

[47] Macfarlane R, Heng DYC, Xie W, Knox JJ, McDermott DF, Rini BI, Kollmannsberger C, Choueiri TK (2012). The impact of kidney function on the outcome of metastatic renal cell carcinoma patients treated with vascular endothelial growth factor-targeted therapy. Cancer.

[48] Masini C, Vitale MG, Maruzzo M, Procopio G, de Giorgi U, Buti S, Rossetti S, Iacovelli R, Atzori F, Cosmai L, Vignani F, Prati G, Scagliarini S, Guida A, Berselli A, Pinto C (2019). Safety and efficacy of pazopanib in first-line metastatic renal-cell carcinoma with or without renal failure: CORE-URO-01 study. Clin Genitourin Cancer.

[49] Kennoki T, Kondo T, Kimata N, Murakami J, Ishimori I, Nakazawa H, Hashimoto Y, Kobayashi H, Iizuka J, Takagi T, Yoshida K, Tanabe K (2011). Clinical results and pharmacokinetics of sorafenib in chronic hemodialysis patients with metastatic renal cell carcinoma in a single center. Jpn J Clin Oncol.

[50] Izzedine H, Etienne-Grimaldi MC, Renée N, Vignot S, Milano G (2009). Pharmacokinetics of sunitinib in hemodialysis. Ann Oncol.

[51] Thiery-Vuillemin A, Orillard E, Mouillet G, Calcagno F, Devillard N, Bouchet S, Royer B (2017). Hemodialysis does not impact axitinib exposure: clinical case of a patient with metastatic renal cell carcinoma. Cancer Chemother Pharmacol.

[52] Noda S, Hira D, Kageyama S, Jo F, Wada A, Yoshida T, Kawauchi A, Morita S-y, Terada T (2016). Pharmacokinetic analysis of a hemodialyzed patient treated with pazopanib. Clin Genitourin Cancer.

[53] Leonetti A, Bersanelli M, Castagneto B, Masini C, Di Meglio G, Pellegrino B, Buti S (2016). Outcome and safety of sorafenib in metastatic renal cell carcinoma dialysis patients: a systematic review. Clin Genitourin Cancer.

[54] Gore ME, Szczylik C, Porta C, Bracarda S, Bjarnason GA, Oudard S, Hariharan S, Lee SH, Haanen J, Castellano D, Vrdoljak E, Schöffski P, Mainwaring P, Nieto A, Yuan J, Bukowski R (2009). Safety and efficacy of sunitinib for metastatic renal-cell carcinoma: an expanded-access trial. Lancet Oncol.

[55] Stadler WM, Figlin RA, McDermott DF, Dutcher JP, Knox JJ, Miller WH, Hainsworth JD, Henderson CA, George JR, Hajdenberg J, Kindwall-Keller TL, Ernstoff MS, Drabkin HA, Curti BD, Chu L, Ryan CW, Hotte SJ, Xia C, Cupit L, Bukowski RM (2010). Safety and efficacy results of the advanced renal cell carcinoma sorafenib expanded access program in North America. Cancer.

[56] Bersanelli M, Facchinetti F, Tiseo M, Maiorana M, Buti S (2016). Pazopanib in renal cell carcinoma dialysis patients: a mini-review and a case report. Curr Drug Targets.

[57] Ishihara H, Takagi T, Kondo T, Yoshida K, Okumi M, Tanabe K (2019). Efficacy and safety of axitinib for metastatic renal cell carcinoma in patients on hemodialysis for end-stage renal disease: case series of eight patients. Int J Urol.

[58] Pham A, Ye D-W, Pal S (2015). Overview and management of toxicities associated with systemic therapies for advanced renal cell carcinoma. Urol Oncol Semin Orig Investig.

[59] Paluri RK, Sonpavde G, Morgan C, Rojymon J, Mar AH, Gangaraju R (2019). Renal toxicity with mammalian target of rapamycin inhibitors: a meta-analysis of randomized clinical trials. Oncol Rev.

[60] Choueiri TK, Je Y, Sonpavde G, Richards CJ, Galsky MD, Nguyen PL, Schutz F, Heng DY, Kaymakcalan MD (2013). Incidence and risk of treatment-related mortality in cancer patients treated with the mammalian target of rapamycin inhibitors. Ann Oncol.

[61] Bertoni E, Bruschi M, Candiano G, Boccardi C, Citti L, Mangraviti S, Rosso G, Larti A, Rosati A, Ghiggeri GM, Salvadori M (2009). Posttransplant proteinuria associated with everolimus. Transpl Proc.

[62] Lieberthal W, Fuhro R, Andry CC, Rennke H, Abernathy VE, Koh JS, Valeri R, Levine JS (2001). Rapamycin impairs recovery from acute renal failure: role of cell-cycle arrest and apoptosis of tubular cells. Am J Physiol Renal Physiol.

[63] Lieberthal W, Fuhro R, Andry C, Patel V, Levine JS (2006). Rapamycin delays but does not prevent recovery from acute renal failure: role of acquired tubular resistance. Transplantation.

[64] Nakagawa S, Nishihara K, Inui K-i, Masuda S (2012). Involvement of autophagy in the pharmacological effects of the mTOR inhibitor everolimus in acute kidney injury. Eur J Pharmacol.

[65] Izzedine H, Escudier B, Rouvier P, Gueutin V, Varga A, Bahleda R, Soria JC (2013). Acute tubular necrosis associated with mTOR inhibitor therapy: a real entity biopsy-proven. Ann Oncol.

[66] Vogelbacher R, Wittmann S, Braun A, Daniel C, Hugo C (2007). The mTOR inhibitor everolimus induces proteinuria and renal deterioration in the remnant kidney model in the rat. Transplantation.

[67] Sarbassov DD, Ali SM, Sengupta S, Sheen J-H, Hsu PP, Bagley AF, Markhard AL, Sabatini DM (2006). Prolonged rapamycin treatment inhibits mTORC2 assembly and Akt/PKB. Mol Cell.

[68] Letavernier E, Legendre C (2008). mToR inhibitors-induced proteinuria: mechanisms, significance, and management. Transplant Rev.

[69] Ha SH, Park JH, Jang HR, Huh W, Lim H-Y, Kim Y-G, Kim DJ, Oh HY, Lee JE (2014). Increased risk of everolimus-associated acute kidney injury in cancer patients with impaired kidney function. BMC Cancer.

[70] Kim KH, Kim JH, Lee JY, Kim HS, Heo SJ, Kim JH, Kim HY, Rha SY (2016). Efficacy and toxicity of mammalian target rapamycin inhibitors in patients with metastatic renal cell carcinoma with renal insufficiency: the Korean Cancer Study Group GU 14–08. Cancer Res Treat.

[71] Lunardi G, Vannozzi MO, Armirotti A, Nicodemo M, Venturini M, Cavallini L (2008) Temsirolimus in patients with renal cancer on hemodialysis. J Clin Oncol 26 (34):5652–5653; author reply 5653–5654. 10.1200/JCO.2008.19.3144 10.1200/JCO.2008.19.3144 18981458

[72] Thiery-Vuillemin A, Curtit E, Maurina T, Montange D, Succi C, Nguyen T, Kim S, Montcuquet P, Pivot X, Royer B (2012). Hemodialysis does not affect everolimus pharmacokinetics: two cases of patients with metastatic renal cell cancer. Ann Oncol.

[73] Guida A, Masini C, Milella M, Di Lorenzo G, Santoni M, Prati V, Porta C, Cosmai L, Donati D, del Giovane C, Mighali P, Sabbatini R (2015). Retrospective analysis on safety and efficacy of everolimus in treatment of metastatic renal cancer patients receiving dialysis. Future Oncol.

[74] Motzer RJ, Escudier B, Oudard S, Hutson TE, Porta C, Bracarda S, Grünwald V, Thompson JA, Figlin RA, Hollaender N, Kay A, for the R-SG (2010). Phase 3 trial of everolimus for metastatic renal cell carcinoma. Cancer.

[75] Escudier B, Porta C, Schmidinger M, Rioux-Leclercq N, Bex A, Khoo V, Grünwald V, Gillessen S, Horwich A (2019). Renal cell carcinoma: ESMO clinical practice guidelines for diagnosis, treatment and follow-up†. Ann Oncol.

[76] Longo DLMD, Postow MAMD, Sidlow RMD, Hellmann MDMD (2018). Immune-related adverse events associated with immune checkpoint blockade. N Engl J Med.

[77] Seethapathy H, Zhao S, Chute DF, Zubiri L, Oppong Y, Strohbehn I, Cortazar FB, Leaf DE, Mooradian MJ, Villani A-C, Sullivan RJ, Reynolds K, Sise ME (2019). The incidence, causes, and risk factors of acute kidney injury in patients receiving immune checkpoint inhibitors. Clin J Am Soc Nephrol.

[78] Manohar S, Kompotiatis P, Thongprayoon C, Cheungpasitporn W, Herrmann J, Herrmann SM (2018). Programmed cell death protein 1 inhibitor treatment is associated with acute kidney injury and hypocalcemia: meta-analysis. Nephrol Dial Transplant.

[79] Motzer RJ, Rini BI, McDermott DF, Arén Frontera O, Hammers HJ, Carducci MA, Salman P, Escudier B, Beuselinck B, Amin A, Porta C, George S, Neiman V, Bracarda S, Tykodi SS, Barthélémy P, Leibowitz-Amit R, Plimack ER, Oosting SF, Redman B, Melichar B, Powles T, Nathan P, Oudard S, Pook D, Choueiri TK, Donskov F, Grimm M-O, Gurney H, Heng DYC, Kollmannsberger CK, Harrison MR, Tomita Y, Duran I, Grünwald V, McHenry MB, Mekan S, Tannir NM (2019). Nivolumab plus ipilimumab versus sunitinib in first-line treatment for advanced renal cell carcinoma: extended follow-up of efficacy and safety results from a randomised, controlled, phase 3 trial. Lancet Oncol.

[80] Cortazar FB, Marrone KA, Troxell ML, Ralto KM, Hoenig MP, Brahmer JR, Le DT, Lipson EJ, Glezerman IG, Wolchok J, Cornell LD, Feldman P, Stokes MB, Zapata SA, Hodi FS, Ott PA, Yamashita M, Leaf DE (2016). Clinicopathological features of acute kidney injury associated with immune checkpoint inhibitors. Kidney Int.

[81] Motzer RJ, Penkov K, Haanen J, Rini B, Albiges L, Campbell MT, Venugopal B, Kollmannsberger C, Negrier S, Uemura M, Lee JL, Vasiliev A, Miller WH, Gurney H, Schmidinger M, Larkin J, Atkins MB, Bedke J, Alekseev B, Wang J, Mariani M, Robbins PB, Chudnovsky A, Fowst C, Hariharan S, Huang B, di Pietro A, Choueiri TK (2019). Avelumab plus axitinib versus sunitinib for advanced renal-cell carcinoma. N Engl J Med.

[82] Powles T, Plimack ER, Soulières D, Waddell T, Stus V, Gafanov R, Nosov D, Pouliot F, Melichar B, Vynnychenko I, Azevedo SJ, Borchiellini D, McDermott RS, Bedke J, Tamada S, Yin L, Chen M, Molife LR, Atkins MB, Rini BI (2020). Pembrolizumab plus axitinib versus sunitinib monotherapy as first-line treatment of advanced renal cell carcinoma (KEYNOTE-426): extended follow-up from a randomised, open-label, phase 3 trial. Lancet Oncol.

[83] Amin A, Plimack ER, Ernstoff MS, Lewis LD, Bauer TM, McDermott DF, Carducci M, Kollmannsberger C, Rini BI, Heng DYC, Knox J, Voss MH, Spratlin J, Berghorn E, Yang L, Hammers HJ (2018). Safety and efficacy of nivolumab in combination with sunitinib or pazopanib in advanced or metastatic renal cell carcinoma: the CheckMate 016 study. J Immunother Cancer.

[84] Lo WK, Yau T, Chang TYA, Chan KW (2020). SAT-024 Glomerulonephritis induced by combination immunotherapy and VEGF inhibition with tyrosine kinase inhibitors (TKI). Kidney Int Rep.

[85] Perazella MA, Shirali AC (2020). Immune checkpoint inhibitor nephrotoxicity: what do we know and what should we do?. Kidney Int.

[86] Cortazar FB, Kibbelaar ZA, Glezerman IG, Abudayyeh A, Mamlouk O, Motwani SS, Murakami N, Herrmann SM, Manohar S, Shirali AC, Kitchlu A, Shirazian S, Assal A, Vijayan A, Renaghan AD, Ortiz-Melo DI, Rangarajan S, Malik AB, Hogan JJ, Dinh AR, Shin DS, Marrone KA, Mithani Z, Johnson DB, Hosseini A, Uprety D, Sharma S, Gupta S, Reynolds KL, Sise ME, Leaf DE (2020). Clinical features and outcomes of immune checkpoint inhibitor-associated AKI: a multicenter study. J Am Soc Nephrol.

[87] Mamlouk O, Selamet U, Machado S, Abdelrahim M, Glass WF, Tchakarov A, Gaber L, Lahoti A, Workeneh B, Chen S, Lin J, Abdel-Wahab N, Tayar J, Lu H, Suarez-Almazor M, Tannir N, Yee C, Diab A, Abudayyeh A (2019). Nephrotoxicity of immune checkpoint inhibitors beyond tubulointerstitial nephritis: single-center experience. J Immunother Cancer.

[88] Shingarev R, Glezerman IG (2019). Kidney complications of immune checkpoint inhibitors: a review. Am J Kidney Dis.

[89] Gupta S, Cortazar FB, Riella LV, Leaf DE (2020). Immune checkpoint inhibitor nephrotoxicity: update 2020. Kidney 360.

[90] Wanchoo R, Karam S, Uppal NN, Barta VS, Deray G, Devoe C, Launay-Vacher V, Jhaveri KD (2017). Adverse renal effects of immune checkpoint inhibitors: a narrative review. Am J Nephrol.

[91] Granier C, De Guillebon E, Blanc C, Roussel H, Badoual C, Colin E, Saldmann A, Gey A, Oudard S, Tartour E (2017). Mechanisms of action and rationale for the use of checkpoint inhibitors in cancer. ESMO Open.

[92] Tivol EA, Borriello F, Schweitzer AN, Lynch WP, Bluestone JA, Sharpe AH (1995). Loss of CTLA-4 leads to massive lymphoproliferation and fatal multiorgan tissue destruction, revealing a critical negative regulatory role of CTLA-4. Immunity.

[93] Nishimura H, Nose M, Hiai H, Minato N, Honjo T (1999). Development of lupus-like autoimmune diseases by disruption of the PD-1 gene encoding an ITIM motif-carrying immunoreceptor. Immunity.

[94] Herrmann SM, Perazella MA (2020). Immune checkpoint inhibitors and immune-related adverse renal events. Kidney Int Rep.

[95] Perazella MA, Sprangers B (2019). AKI in patients receiving immune checkpoint inhibitors. Clin J Am Soc Nephrol.

[96] Brahmer JR, Lacchetti C, Schneider BJ, Atkins MB, Brassil KJ, Caterino JM, Chau I, Ernstoff MS, Gardner JM, Ginex P, Hallmeyer S, Holter Chakrabarty J, Leighl NB, Mammen JS, McDermott DF, Naing A, Nastoupil LJ, Phillips T, Porter LD, Puzanov I, Reichner CA, Santomasso BD, Seigel C, Spira A, Suarez-Almazor ME, Wang Y, Weber JS, Wolchok JD, Thompson JA (2018). Management of immune-related adverse events in patients treated with immune checkpoint inhibitor therapy: american society of clinical oncology clinical practice guideline. J Clin Oncol.

[97] Haanen JBAG, Carbonnel F, Robert C, Kerr KM, Peters S, Larkin J, Jordan K (2017). Management of toxicities from immunotherapy: ESMO clinical practice guidelines for diagnosis, treatment and follow-up. Ann Oncol.

[98] Koda R, Watanabe H, Tsuchida M, Iino N, Suzuki K, Hasegawa G, Imai N, Narita I (2018). Immune checkpoint inhibitor (nivolumab)-associated kidney injury and the importance of recognizing concomitant medications known to cause acute tubulointerstitial nephritis: a case report. BMC Nephrol.

[99] Bajaj G, Wang X, Agrawal S, Gupta M, Roy A, Feng Y (2017). Model-based population pharmacokinetic analysis of nivolumab in patients with solid tumors. CPT Pharmacomet Syst Pharmacol.

[100] Balar AV, Galsky MD, Rosenberg JE, Powles T, Petrylak DP, Bellmunt J, Loriot Y, Necchi A, Hoffman-Censits J, Perez-Gracia JL, Dawson NA, van der Heijden MS, Dreicer R, Srinivas S, Retz MM, Joseph RW, Drakaki A, Vaishampayan UN, Sridhar SS, Quinn DI, Durán I, Shaffer DR, Eigl BJ, Grivas PD, Yu EY, Li S, Kadel EE, Boyd Z, Bourgon R, Hegde PS, Mariathasan S, Thåström A, Abidoye OO, Fine GD, Bajorin DF (2017). Atezolizumab as first-line treatment in cisplatin-ineligible patients with locally advanced and metastatic urothelial carcinoma: a single-arm, multicentre, phase 2 trial. Lancet.

[101] Tachibana H, Kondo T, Ishihara H, Takagi T, Tanabe K (2019). Safety and efficacy of nivolumab in patients with metastatic renal cell carcinoma and end-stage renal disease at 2 centers. Clin Genitourin Cancer.

[102] Capasso A, Benigni A, Capitanio U, Danesh FR, Di Marzo V, Gesualdo L, Grandaliano G, Jaimes EA, Malyszko J, Perazella MA, Qian Q, Ronco P, Rosner MH, Trepiccione F, Viggiano D, Zoccali C, Capasso G, International Conference on Onco-Nephrology P (2019). Summary of the International Conference on Onco-Nephrology: an emerging field in medicine. Kidney Int.

[103] Malyszko J, Tesarova P, Capasso G, Capasso A (2020). The link between kidney disease and cancer: complications and treatment. Lancet.

[104] Motzer RJ, Hutson TE, Olsen MR, Hudes GR, Burke JM, Edenfield WJ, Wilding G, Agarwal N, Thompson JA, Cella D, Bello A, Korytowsky B, Yuan J, Valota O, Martell B, Hariharan S, Figlin RA (2012). Randomized phase II trial of sunitinib on an intermittent versus continuous dosing schedule as first-line therapy for advanced renal cell carcinoma. J Clin Oncol.

[105] Motzer RJ, Rini BI, Bukowski RM, Curti BD, George DJ, Hudes GR, Redman BG, Margolin KA, Merchan JR, Wilding G, Ginsberg MS, Bacik J, Kim ST, Baum CM, Michaelson MD (2006). Sunitinib in patients with metastatic renal cell carcinoma. JAMA J Am Med Assoc.

[106] Motzer RJ, Barrios CH, Kim TM, Falcon S, Cosgriff T, Harker WG, Pittman KB, Sabbatini R, Rha SY, Flaig TW, Page RD, Bavbek SE, Beck JT, Patel PM, Schiff E, Vaury A, Niolat J, Gogov S, Anak O, Knox J (2013). Record-3: Phase II randomized trial comparing sequential first-line everolimus (EVE) and second-line sunitinib (SUN) versus first-line SUN and second-line EVE in patients with metastatic renal cell carcinoma (mRCC). J Clin Oncol.

[107] Hutson TE, Davis ID, Machiels JP, De Souza PL, Rottey S, Hong BF, Epstein RJ, Baker KL, McCann L, Crofts T, Pandite L, Figlin RA (2010). Efficacy and safety of pazopanib in patients with metastatic renal cell carcinoma. J Clin Oncol.

[108] Hainsworth JD, Rubin MS, Arrowsmith ER, Khatcheressian J, Crane EJ, Franco LA (2013). Pazopanib as second-line treatment after sunitinib or bevacizumab in patients with advanced renal cell carcinoma: a Sarah Cannon Oncology Research Consortium Phase II Trial. Clin Genitourin Cancer.

[109] Sternberg CN, Davis ID, Mardiak J, Szczylik C, Lee E, Wagstaff J, Barrios CH, Salman P, Gladkov OA, Kavina A, Zarba JJ, Chen M, McCann L, Pandite L, Roychowdhury DF, Hawkins RE (2010). Pazopanib in locally advanced or metastatic renal cell carcinoma: results of a randomized phase III trial. J Clin Oncol.

[110] Sternberg CN, Hawkins RE, Wagstaff J, Salman P, Mardiak J, Barrios CH, Zarba JJ, Gladkov OA, Lee E, Szczylik C, McCann L, Rubin SD, Chen M, Davis ID (2013). A randomised, double-blind phase III study of pazopanib in patients with advanced and/or metastatic renal cell carcinoma: final overall survival results and safety update. Eur J Cancer.

[111] Escudier B, Eisen T, Stadler WM, Szczylik C, Oudard S, Siebels M, Negrier S, Chevreau C, Solska E, Desai AA, Rolland F, Demkow T, Hutson TE, Gore M, Freeman S, Schwartz B, Shan M, Simantov R, Bukowski RM, Group TS (2007). Sorafenib in advanced clear-cell renal-cell carcinoma. N Engl J Med.

[112] Escudier B, Eisen T, Stadler WM, Szczylik C, Oudard S, Staehler M, Negrier S, Chevreau C, Desai AA, Rolland F, Demkow T, Hutson TE, Gore M, Anderson S, Hofilena G, Shan M, Pena C, Lathia C, Bukowski RM (2009). Sorafenib for treatment of renal cell carcinoma: Final efficacy and safety results of the phase III treatment approaches in renal cancer global evaluation trial. J Clin Oncol.

[113] Hutson TE, Escudier B, Esteban E, Bjarnason GA, Lim HY, Pittman KB, Senico P, Niethammer A, Lu DR, Hariharan S, Motzer RJ (2014). Randomized phase III trial of temsirolimus versus sorafenib as second-line therapy after sunitinib in patients with metastatic renal cell carcinoma. J Clin Oncol.

[114] Rixe O, Bukowski RM, Michaelson MD, Wilding G, Hudes GR, Bolte O, Motzer RJ, Bycott P, Liau KF, Freddo J, Trask PC, Kim S, Rini BI (2007). Axitinib treatment in patients with cytokine-refractory metastatic renal-cell cancer: a phase II study. Lancet Oncol.

[115] Eto M, Uemura H, Tomita Y, Kanayama H, Shinohara N, Kamei Y, Fujii Y, Umeyama Y, Ozono S, Naito S, Akaza H, Japan Axitinib Phase IISG (2014). Overall survival and final efficacy and safety results from a Japanese phase II study of axitinib in cytokine-refractory metastatic renal cell carcinoma. Cancer Sci.

[116] Motzer RJ, Escudier B, Tomczak P, Hutson TE, Michaelson MD, Negrier S, Oudard S, Gore ME, Tarazi J, Hariharan S, Chen C, Rosbrook B, Kim S, Rini BI (2013). Axitinib versus sorafenib as second-line treatment for advanced renal cell carcinoma: overall survival analysis and updated results from a randomised phase 3 trial. Lancet Oncol.

[117] Qin S, Bi F, Jin J, Cheng Y, Guo J, Ren X, Huang Y, Tarazi J, Tang J, Chen C, Kim S, Ye D (2015). Axitinib versus sorafenib as a second-line therapy in Asian patients with metastatic renal cell carcinoma: results from a randomized registrational study. Onco Targets Ther.

[118] Hutson TE, Al-Shukri S, Stus VP, Lipatov ON, Shparyk Y, Bair AH, Rosbrook B, Andrews GI, Vogelzang NJ (2017). axitinib versus sorafenib in first-line metastatic renal cell carcinoma: overall survival from a randomized phase III trial. Clin Genitourin Cancer.

[119] Hutson TE, Lesovoy V, Al-Shukri S, Stus VP, Lipatov ON, Bair AH, Rosbrook B, Chen C, Kim S, Vogelzang NJ (2013). Axitinib versus sorafenib as first-line therapy in patients with metastatic renal-cell carcinoma: a randomised open-label phase 3 trial. Lancet Oncol.

[120] George DJ, Hessel C, Halabi S, Michaelson MD, Hahn O, Walsh M, Picus J, Small EJ, Dakhil S, Feldman DR, Mangeshkar M, Scheffold C, Morris MJ, Choueiri TK (2019). Cabozantinib versus sunitinib for untreated patients with advanced renal cell carcinoma of intermediate or poor risk: subgroup analysis of the alliance A031203 CABOSUN trial. Oncologist.

[121] Choueiri TK, Halabi S, Sanford BL, Hahn O, Michaelson MD, Walsh MK, Feldman DR, Olencki T, Picus J, Small EJ, Dakhil S, George DJ, Morris MJ (2017). Cabozantinib versus sunitinib as initial targeted therapy for patients with metastatic renal cell carcinoma of poor or intermediate risk: the alliance A031203 CABOSUN trial. J Clin Oncol.

[122] Choueiri TK, Hessel C, Halabi S, Sanford B, Michaelson MD, Hahn O, Walsh M, Olencki T, Picus J, Small EJ, Dakhil S, Feldman DR, Mangeshkar M, Scheffold C, George D, Morris MJ (2018). Cabozantinib versus sunitinib as initial therapy for metastatic renal cell carcinoma of intermediate or poor risk (alliance A031203 CABOSUN randomised trial): progression-free survival by independent review and overall survival update. Eur J Cancer.

[123] Choueiri TK, Escudier B, Powles T, Tannir NM, Mainwaring PN, Rini BI, Hammers HJ, Donskov F, Roth BJ, Peltola K, Lee JL, Heng DYC, Schmidinger M, Agarwal N, Sternberg CN, McDermott DF, Aftab DT, Hessel C, Scheffold C, Schwab G, Hutson TE, Pal S, Motzer RJ, Investigators M (2016). Cabozantinib versus everolimus in advanced renal cell carcinoma (METEOR): final results from a randomised, open-label, phase 3 trial. Lancet Oncol.

[124] Choueiri TK, Escudier B, Powles T, Mainwaring PN, Rini BI, Donskov F, Hammers H, Hutson TE, Lee JL, Peltola K, Roth BJ, Bjarnason GA, Geczi L, Keam B, Maroto P, Heng DY, Schmidinger M, Kantoff PW, Borgman-Hagey A, Hessel C, Scheffold C, Schwab GM, Tannir NM, Motzer RJ, Investigators M (2015). Cabozantinib versus everolimus in advanced renal-cell carcinoma. N Engl J Med.

[125] Pfizer Inc.. Sutent (sunitinib) [package insert]. U.S. Food and Drug Administration website. https://www.accessdata.fda.gov/drugsatfda_docs/label/2020/021938s037lbl.pdf. Revised Aug 2020. Accessed 20 Oct 2020

[126] Bayer HealthCare Pharmaceuticals Inc.. Nexavar (sorafenib) [package insert]. U.S. Food and Drug Administration website. https://www.accessdata.fda.gov/drugsatfda_docs/label/2020/021923s024lblrpl.pdf. Revised June 2020. Accessed 2020 Oct 2020

[127] Novartis Pharmaceuticals Corporation. Votrient (pazopanib) [package insert]. U.S. Food and Drug Administration website. https://www.accessdata.fda.gov/drugsatfda_docs/label/2020/022465s029lbl.pdf. Revised Aug 2020. Accessed 20 Oct 2020

[128] Pfizer Inc.. Inlyta (axitinib) [package insert]. U.S. Food and Drug Administration website. https://www.accessdata.fda.gov/drugsatfda_docs/label/2020/202324s011lbl.pdf. Revised June 2020. Accessed 20 Oct 2020

[129] Novartis Pharmaceuticals Corporation. Afinitor (everolimus) [package insert]. U.S. Food and Drug Administration website. https://www.accessdata.fda.gov/drugsatfda_docs/label/2020/022334s044,203985s016lbl.pdf. Revised Feb 2020. Accessed 20 Oct 2020

[130] Wyeth Pharmaceuticals Inc.. Torisel (temsirolimus) [package insert]. U.S. Food and Drug Administration website. https://www.accessdata.fda.gov/drugsatfda_docs/label/2018/022088s021s023lbl.pdf. Revised Mar 2018. Accessed 20 Oct 2020

[131] Bristol-Myers Squibb Company. Yervoy (ipilimumab) [package insert]. U.S. Food and Drug Administration website. https://www.accessdata.fda.gov/drugsatfda_docs/label/2020/125377s115lbl.pdf. Revised Oct 2020. Accessed 20 Oct 2020

[132] Bristol-Myers Squibb Company. Opdivo (nivolumab) [package insert]. U.S. Food and Drug Administration website. https://www.accessdata.fda.gov/drugsatfda_docs/label/2020/125554s089lbl.pdf. Revised Oct 2020. Accessed 20 Oct 2020

[133] Merck Sharp & Dohme Corp.. Keytruda (pembrolizumab) [package insert]. U.S. Food and Drug Administration website. https://www.accessdata.fda.gov/drugsatfda_docs/label/2020/125514s085lbl.pdf. Revised Oct 2020. Accessed 20 Oct 2020

[134] Genentech Inc.. Tecentriq (atezolizumab) [package insert]. U.S. Food and Drug Administration website. https://www.accessdata.fda.gov/drugsatfda_docs/label/2020/761034s029lbl.pdf. Revised Sept 2020. Accessed 20 Oct 2020

[135] Josephs D, Hutson TE, Cowey CL, Pickering LM, Larkin JM, Gore ME, Van Hemelrijck M, McDermott DF, Powles T, Chowdhury P, Karapetis C, Harper PG, Choueiri TK, Chowdhury S (2011). Efficacy and toxicity of sunitinib in patients with metastatic renal cell carcinoma with severe renal impairment or on haemodialysis. BJU Int.

[136] Casper J, Goebel D, Gruenwald V, Flörcken A, Mueller D, Toussaint K, Metzner B (2011). Efficacy and safety of sunitinib in patients with metastatic renal cell carcinoma on hemodialysis. J Clin Oncol.

[137] Masini C, Sabbatini R, Porta C, Procopio G, Di Lorenzo G, Onofri A, Buti S, Iacovelli R, Invernizzi R, Moscetti L, Aste MG, Pagano M, Grosso F, Lucia Manenti A, Ortega C, Cosmai L, Del Giovane C, Conte PF (2012). Use of tyrosine kinase inhibitors in patients with metastatic kidney cancer receiving haemodialysis: a retrospective Italian survey. BJU Int.

[138] Shetty AV, Matrana MR, Atkinson BJ, Flaherty AL, Jonasch E, Tannir NM (2014). Outcomes of patients with metastatic renal cell carcinoma and end-stage renal disease receiving dialysis and targeted therapies: a single institution experience. Clin Genitourin Cancer.

[139] Czarnecka AM, Kawecki M, Lian F, Korniluk J, Szczylik C (2015). Feasibility, efficacy and safety of tyrosine kinase inhibitor treatment in hemodialyzed patients with renal cell cancer: 10 years of experience. Future Oncol.

[140] Omae K, Kondo T, Kennoki T, Takagi T, Iizuka J, Kobayashi H, Hashimoto Y, Tanabe K (2016). Efficacy and safety of sorafenib for treatment of Japanese metastatic renal cell carcinoma patients undergoing hemodialysis. Int J Clin Oncol.

[141] Syrios J, Kechagias G, Tsavaris N (2013). Treatment of patients with metastatic renal cell carcinoma undergoing hemodialysis: case report of two patients and short literature review. BMC Nephrol.

[142] Miyake H, Harada K, Kusuda Y, Fujisawa M (2013). Efficacy and safety of temsirolimus in Japanese patients with metastatic renal cell carcinoma on hemodialysis. Int J Clin Oncol.

[143] Omae K, Kondo T, Takagi T, Iizuka J, Kobayashi H, Hashimoto Y, Tanabe K (2016). Use of mammalian target of rapamycin inhibitors after failure of tyrosine kinase inhibitors in patients with metastatic renal cell carcinoma undergoing hemodialysis: a single-center experience with four cases. Hemodial Int.

[144] Carlo MI, Feldman DR (2016). Response to nivolumab in a patient with metastatic clear cell renal cell carcinoma and end-stage renal disease on dialysis. Eur Urol.

[145] Tabei T, Natsume I, Kobayashi K (2017). Successful treatment of metastatic clear cell carcinoma with nivolumab in a patient receiving dialysis treatment. Int J Urol.

[146] Ansari J, Ali M, Farrag A, Ali AM, Alhamad A (2018). Efficacy of nivolumab in a patient with metastatic renal cell carcinoma and end-stage renal disease on dialysis: case report and literature review. Case Rep Immunol.

[147] Cheun H, Kim M, Lee H, Oh KH, Keam B (2019). Safety and efficacy of immune checkpoint inhibitors for end-stage renal disease patients undergoing dialysis: a retrospective case series and literature review. Investig New Drugs.

[148] Vitale MG, Baldessari C, Milella M, Buti S, Militello AM, Di Girolamo S, Fornarini G, Perri G, Basso U, Maruzzo M, Porta C, Cosmai L, Pipitone S, Cerma K, Cascinu S, Sabbatini R (2019). Immunotherapy in dialysis-dependent cancer patients: our experience in patients with metastatic renal cell carcinoma and a review of the literature. Clin Genitourin Cancer.

